# Evaluation of Turbidity and Color Removal in Water Treatment: A Comparative Study between *Opuntia ficus-indica* Fruit Peel Mucilage and FeCl_3_

**DOI:** 10.3390/polym15010217

**Published:** 2022-12-31

**Authors:** Maria Carolina Otálora, Andrea Wilches-Torres, Carlos Rafael Lara, Jovanny A. Gómez Castaño, Gabriel Ricardo Cifuentes

**Affiliations:** 1 Grupo de Investigación en Ciencias Básicas (NÚCLEO), Facultad de Ciencias e Ingeniería, Universidad de Boyacá, Tunja 150003, Colombia; 2 Grupo Gestión de Recursos Hídricos, Facultad de Ciencias e Ingeniería, Universidad de Boyacá, Tunja 150003, Colombia; 3 Grupo Química-Física Molecular y Modelamiento Computacional (QUIMOL^®^), Escuela de Ciencias Químicas, Universidad Pedagógica y Tecnológica de Colombia, Sede Tunja, Avenida Central del Norte, Tunja 150003, Colombia

**Keywords:** *Opuntia ficus-indica*, water/wastewater treatment, coagulation/flocculation process, mucilage, biocoagulant

## Abstract

Natural coagulants derived from by-products have gained popularity as sustainable alternatives to inorganic coagulants in water/wastewater treatment due to their abundant availability, biodegradability, low cost, easy disposal and low sludge volumes. In this study, the mucilage obtained from the peel of *Opuntia ficus-indica* fruit was evaluated as a biocoagulant for treating synthetic turbid water and compared with a traditional chemical coagulant (FeCl_3_). The effects of coagulant dosage and pH on the turbidity and color-removal efficiency of synthetic turbid water were analyzed. To estimate the coagulation mechanism, the flocs produced under optimal values were characterized structurally (FTIR and zeta potential) and morphologically (SEM). The optimal condition for the removal of turbidity and color was a coagulant dose of 12 mg/L at pH 13. For the optimal values, the biocoagulant and the FeCl_3_ presented a maximum removal of 82.7 ± 3.28% and 94.63 ± 0.98% for turbidity and 71.82 ± 2.72% and 79.94 ± 1.77% for color, respectively. The structure and morphology of the flocs revealed that the coagulation mechanism of the mucilage was adsorption and bridging, whereas that of FeCl_3_ was charge neutralization. The results obtained showed that the mucilage could be used as an alternative coagulant to replace FeCl_3_.

## 1. Introduction

Due to its high renewal capacity, biodegradability, lower sludge production, non-toxicity and profitability, the use of coagulants based on natural polysaccharides has emerged as a promising alternative to replace traditional inorganic coagulants, such as ferric sulfate (Fe_2_(SO_4_)_3_), ferric chloride (FeCl_3_), aluminum sulfate (Al_2_(SO_4_)_3_), aluminum chloride (AlCl_3_) and titanium chloride (TiCl), used in water and wastewater treatment [1]. Inorganic coagulants operate through a mechanism of destabilization of dispersed organic matter by the formation of microflocs, followed by aggregation and subsequent sedimentation by gravity [2]. However, these coagulants have drawbacks associated with their high costs, adverse effects on human health and production of large amounts of sludge with potential risks to the environment [3]. In contrast, the use of mucilaginous biomaterials extracted from agricultural by-products, such as the peels of cactaceous plant fruits, represents a more sustainable and environmentally friendly coagulant option [4].

The use of peels as a natural raw material has been widely evaluated in coagulation/flocculation processes. For example, Daverey et al. [5] demonstrated that aqueous extracts of *Musa paradisiaca* (banana) peels are better flocculants than *Dolichos lablab* (Indian bean) seeds, and that they can be used to remove turbidity in water treatment. Likewise, Afolabi et al. [6] determined that banana peel can be used in the removal of Pb(II) from an aqueous solution. Hodur et al. [7] investigated the effect of pomegranate peel treatment on ammonium nitrogen removal from milking parlor wastewater. Ibisi et al. [8] examined the use of *Musa paradisiaca* peels as natural coagulants to remove Pb^2+^ and Cd^2+^ (toxic metal ions) from contaminated water. Oyewo et al. [9] investigated the effect of nanosorbent treatment of banana peels on the removal of radioactive minerals (uranium and thorium) from mining water.

In Latin America, the consumption of fresh fruits of *Opuntia ficus-indica* (OFI) results in the accumulation of large quantities of peels, which represent an underutilized deposit of mucilage rich in polysaccharides [10,11,12]. This mucilage is an anionic polyelectrolyte heteropolysaccharide, consisting of arabinose (34%), galactose (54%) and xylose (10%) as predominant monosaccharides [12,13], highly hydrophilic, soluble and dispersible in water [14]. We have recently extracted and physicochemically characterized the mucilage from the peel of the OFI fruit, thus finding adequate molecular properties for the use of this biopolymer as a potential natural coagulant in water or wastewater treatment [4]. As far as we know, there are no reports on the use of OFI fruit peel mucilage as a biocoagulant for water clarification. Consequently, this work aimed to determine the coagulation efficiency of the mucilage obtained from OFI fruit peels for the removal of turbidity and color from synthetic turbid waters. The efficiency of this biocoagulant was compared with the removal capacity using ferric chloride (FeCl_3_) as a traditional inorganic coagulant. To elucidate the coagulation mechanism in each case, the flocs obtained under optimal pH and coagulant concentration values were structurally analyzed using FTIR spectroscopy and zeta potential, and morphologically using scanning electron microscopy (SEM). Therefore, this study of the efficiency of the mucilage obtained from OFI fruit peels for the removal of turbidity and color as a sustainable coagulant, and as a replacement for commercial inorganic coagulants used in water clarification, emphasizes the novelty of the current work.

## 2. Materials and Methods

### 2.1. Chemical Reagents and Synthetic Turbid Water

Kaolin (99 wt%) and sodium chloride (NaCl, 96 wt%) were purchased from Merck (Darmstadt, Germany). A 1.2% chemical coagulant stock solution was prepared using ferric chloride (FeCl_3_, 96 wt%) purchased from Sigma-Aldrich (Markham, Canada). Sodium hydroxide (NaOH, 96 wt%) and hydrochloric acid (HCl, 1.179 g·cm^−3^) were purchased from Sigma-Aldrich (Markham, Canada) were used to adjust the pH of the synthetic turbid water.

The synthetic turbid water stock solution was prepared by adding 1.0 g kaolin and 7.0 g of NaCl powder to 14 L of deionized water. The suspension was stirred at 400 rpm for 1 h using a magnetic stirrer (C-MAG HS 7 S000, IKA, Staufen im Breisgau, Germany) at 18 °C until a uniform dispersion of the particles was obtained. The synthetic dispersion presented a pH of 7.85 ± 0.09 and a conductivity of 147 ± 0.01 µS/cm, parameters that were measured using a portable multiparameter meter (HACH HQ 40d, Loveland, CO, USA). The turbidity obtained was 54.33 NTU ± 1.51 and the color 264 ± 8.54, parameters determined using a turbidimeter (2100Q, HACH, Loveland, CO, USA) and a Hach DR 2800 spectrophotometer, respectively.

### 2.2. OFI Mucilage Biocoagulant

Fresh peels of *Opuntia ficus-indica* (OFI) fruits were collected from local food restaurants in the city of Tunja, Boyacá, Colombia. The mucilage extraction was carried out according to the methodology reported by Otálora et al. [4]. Small pieces of OFI fruit peels were placed in a 100 mL beaker, to which distilled water at room temperature was added to a ratio of 1:2 *w*/*v* (peel:water), and left for 12 h. The hydrated peels were manually squeezed to extract the gel. To the obtained gel, 95% ethanol was added in a ratio of 3:1 (ethanol:gel) at 18 °C, and the mixture was allowed to stand for 15 min without stirring until the formation of a milky-white supernatant corresponding to the mucilage. The OFI fruit peel mucilage was collected and then dried in an oven at 50 °C for 3 h. The dried material was manually macerated in a porcelain mortar and subsequently sieved through a 60 mesh until a fine powder was obtained (standard granulometry ≤ 250 μm). The powdered mucilage, used as a natural coagulant, was placed in high-density polyethylene bags and stored in a desiccator at room temperature with 30% relative humidity until use.

### 2.3. Coagulation/Flocculation Process

**Figure 1:**
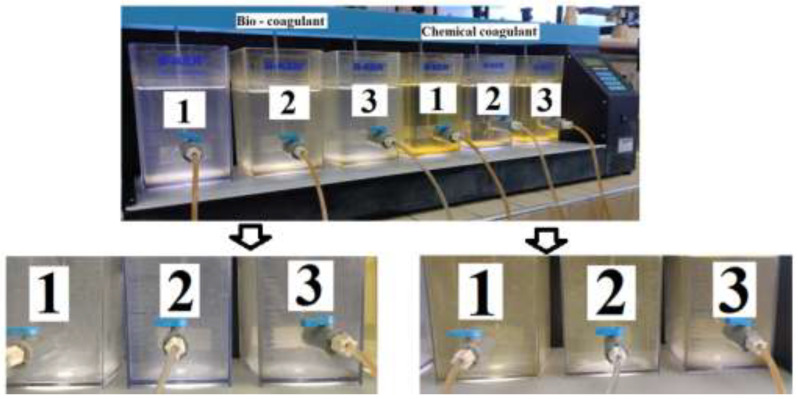
Experimental setup used for the coagulation process using OFI peel mucilage (**left**) and FeCl_3_ (**right**). Labels 1 to 3 refer to the measurements made in triplicate in each case.

Comparative evaluation of the turbidity- and color-removal capacity in synthetic turbid water using both OFI’s natural coagulant and the traditional chemical coagulant (FeCl_3_) was performed using a jar apparatus (Phipps & Bird PB-900, Richmond, VA, USA). This equipment supports six vessels of up to 2000 mL capacity and has an automatic controller that allows programming of the time and speed for fast and slow mixes. All experiments were performed at room temperature (18 ± 1 °C). The initial concentration of the coagulants was set at 1.2 g/100 mL of deionized water. The determination of the optimal dose of coagulants was determined at a pH value of 7.85 ± 0.09 (pH synthetic turbid water) by adding six doses in the range of 6 to 36 mg/L. For the determined optimal coagulant dose, the effects of pH in the range of 1 to 13 were studied. The pH of the water was controlled by adding 1.0 M HCl and 1.0 M NaOH. The suspensions were first stirred at 100 rpm for 1 min of rapid mixing to disperse the coagulant and destabilize the colloidal particles, then stirring was performed at 70 rpm for 20 min of slow mixing to bring the destabilized particles into contact and thus form large flocs. The mixtures were allowed to settle for 20 min and the supernatant was used in the analysis of the water-quality parameters. The analyses of the turbidity and color parameters, using a biocoagulant (peel mucilage) and chemical coagulant (ferric chloride), were carried out in triplicate. Figure 1 shows photographs of the main stage involved in the coagulation process using biocoagulant and chemical coagulant.

Synthetic turbid water samples treated with biocoagulant (OFI fruit peel mucilage) and chemical coagulant (FeCl_3_) were analyzed according to the Standard Methods for the Examination of Water and Wastewater [15] using the following water-quality parameters: color using a spectrophotometer (Hach DR 2800, Loveland, CO, USA) at a wavelength of 455 nm and turbidity using a turbidimeter (2100Q, HACH, Loveland, CO, USA).

The removal efficiency (*R*, %) was calculated using Equation (1).

(1) $$R=\frac{Y_{0}-Y}{Y_{0}}\times \;100,$$

where *Y*_0_ and *Y* were the initial and final values, respectively, in each turbidity- and color-removal evaluation.

### 2.4. Floc Characterization

After the coagulation/flocculation process, an adequate amount of flocculated material was collected and dried in an oven at 50 °C for 24 h. These flocs were characterized structurally using Fourier Transform Infrared spectroscopy (FTIR) and zeta potential and morphologically using scanning electron microscopy (SEM).

#### 2.4.1. Fourier-Transform Infrared (FTIR) Spectroscopy

FTIR spectra of flocs formed under optimum values of coagulants were measured on a Bruker Alpha ECO-ATR spectrophotometer (Bruker, Germany) in the range of 4000–500 cm^−1^ with a resolution of 4.0 cm^−1^ and 24 cumulative scans using the attenuated total reflectance (ATR) technique.

#### 2.4.2. Zeta Potential

The zeta potential of flocs formed under optimum coagulant values was measured using a NanoPlusTM 3 Particle Size Zeta Potential Analyzer (Norcross, GA, USA) at 25 °C and pH 5.33. Powdered flocs were dispersed in 100 mL of ultrapure deionized water using a magnetic stirrer (C-MAG HS 7 S000, IKA, Staufen im Breisgau, Germany) at 8000 rpm for 6 h at room temperature. Results were recorded as the average of triple replicates ± standard deviation.

#### 2.4.3. Scanning Electron Microscopy (SEM)

The microscopic morphology of flocs formed under optimum values of coagulant concentration and water pH was evaluated with scanning electron microscopy (SEM) using EVO MA 10-Carl Zeiss equipment (Oberkochen, Germany) operating at 20 kV. All samples were coated using gold–palladium sputtering before their examination.

### 2.5. Statistical Analysis

Synthetic turbid water parameters, water-quality parameters (turbidity and color), and zeta potential of flocs formed after the coagulation/flocculation process were reported as the mean ± standard deviation (*n* = 3). Data were analyzed using analysis of variance (ANOVA) and means were compared using Fisher’s Least Significant Differences Test (*p* < 0.05).

## 3. Results and Discussion

### 3.1. Dose-Dependent Coagulation Efficiency

**Figure 2:**
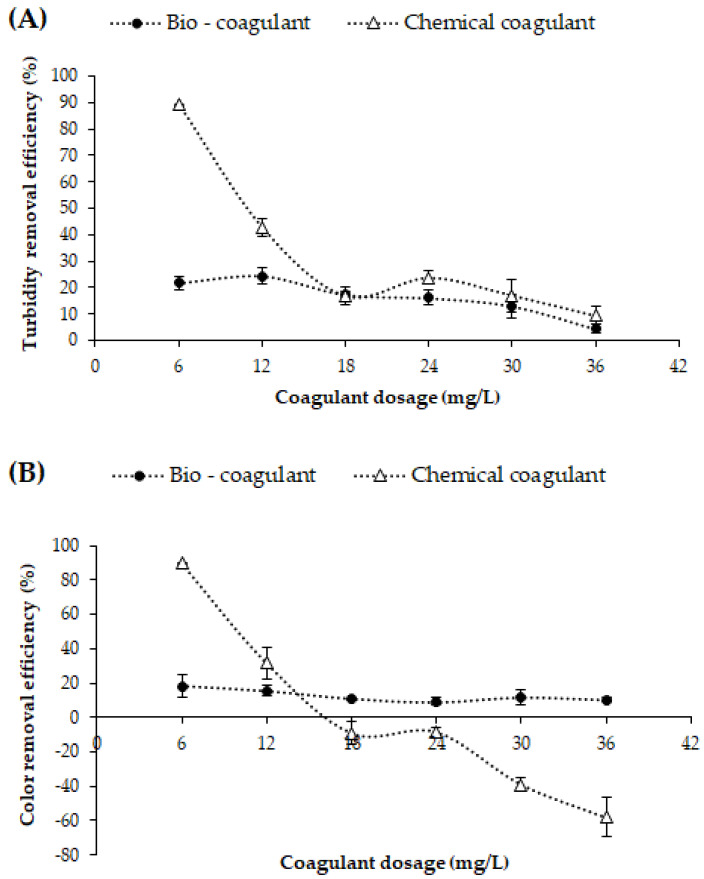
Effects of the biocoagulant (OFI fruit peel mucilage) and chemical coagulant (FeCl_3_) dose on the turbidity-removal efficiency (%) (**A**) and the color-removal efficiency (%) (**B**) at an adjusted pH of 7.85 ± 0.09.

The effect of the dosage of coagulant agents (mucilage and FeCl_3_) in the range of 6 to 36 mg/L at a fixed pH of 7.85 ± 0.09 (synthetic turbid water pH) on the evolution of turbidity and color removal is shown in Figure 2. The results reveal that the maximum removal of turbidity was 24.35 ± 3.13% and of color, 15.44 ± 3.32% (Figure 2a,b), achieved using 12 mg/L of biocoagulant. Moreover, the turbidity- and color-removal efficiency was stable in the dose range. These results agree with those reported by Freitas et al. [16] using okra mucilage in industrial textile wastewater. This result denotes an asymptotic trend of particle removal as the mucilage dose increases, which can be attributed to a process of early coating of the particle surface by the soluble polysaccharides of the mucilage. Such a coating rapidly decreases the number of active adsorption sites and sorption through hydrogen bonding does not occur efficiently; that is, the process of formation of colloidal particles/polysaccharide chain and colloidal particles/polysaccharide chain/complexes is compromised [17,18,19].

In contrast, as evidenced in Figure 2a,b, with increasing FeCl_3_ dosage (>12 mg/L) a decrease in color- and turbidity-removal efficiency was observed, possibly because at low pH (7.85 ± 0. 09) lower doses of chemical coagulant are needed to initiate charge neutralization and precipitation [20] and at higher dosages charge reversal and restabilization take place. Wang et al. [21] determined that the charge-neutralization mechanism is the main mechanism driving the coagulation process when low-dose inorganic coagulants are used. Therefore, the biocoagulant and chemical coagulant dose of 12 mg/L was maintained as the coagulant dose during the investigation due to the destabilization of the colloidal system.

### 3.2. Effect of pH on Coagulation Efficiency

**Figure 3:**
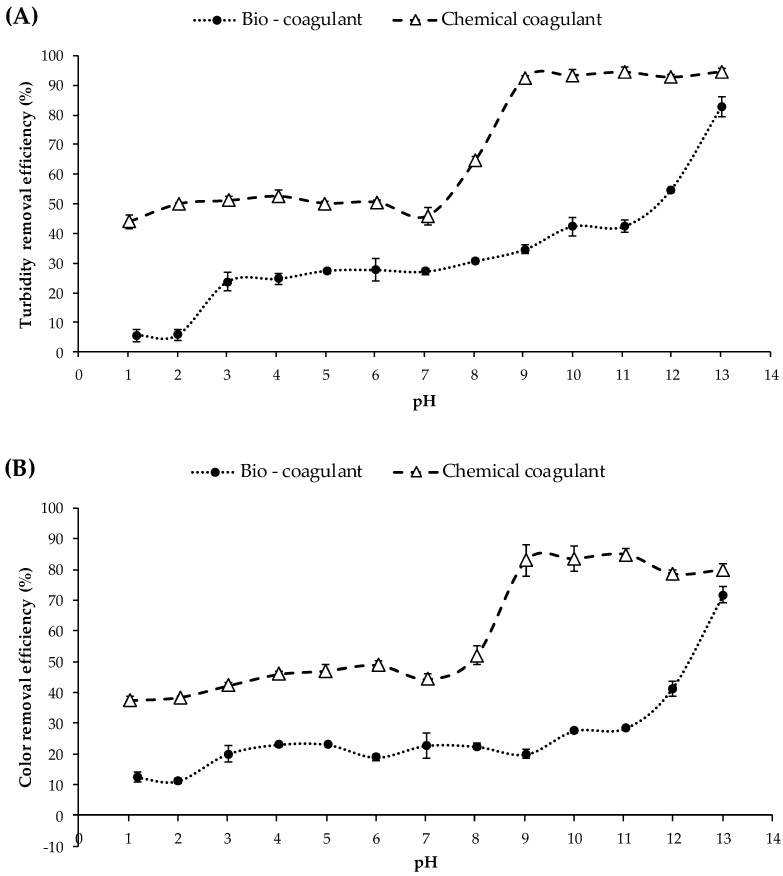
Turbidity- (**A**) and color- (**B**) removal efficiencies at different pH values of synthetic turbid water using a fixed dose of biocoagulant (OFI fruit peel mucilage) and chemical coagulant (ferric chloride) of 12 mg/L.

The effect of synthetic turbid water pH (range 1 to 13) on the evolution of turbidity and color removal using a fixed dose of 12 mg/L OFI peel mucilage or ferric chloride is shown in Figure 3. The efficiency of coagulation gradually increased with increasing pH values until maximum removal at pH = 13 was reached.

As evidenced in Figure 3a,b, synthetic turbid water treated with the natural coagulant presented an evolution of turbidity and color removal between 5 and 27% in the range of pH values from 1 to 9. This behavior was attributed to the fact that at acidic and/or neutral pH values, the ionization reaction of the hydroxyl and carboxyl groups (active sites of galacturonic acid) of the mucilage, as well as the hydrolysis of the glycosidic bonds of the polysaccharide chains, can cause an adsorption process and low bridging between the mucilage molecules and the colloidal particles present in the water sample. However, the synthetic turbid water at pH values between 10 and 13 exhibited turbidity and color removal between 30 and 82% using OFI fruit peel mucilage (Figure 3a,b). In general, this behavior was mainly attributed to a deprotonation reaction of the functional groups of the mucilage, which increases the adsorption sites available on the mucilage molecules leading to greater removal of colloidal particles dispersed in water [22]. The high turbidity and color removal of ≈82% at pH 13 could be attributed to the complex structure of the OFI peel mucilage, which may contain amphoteric ions [23]. A similar result was reported by Chong and Kiew [24], who estimated that banana peel3had its best impurity removal at pH 12. Likewise, Wan et al. [25] estimated that the coagulation capacity of *Opuntia ficus-indica* is greater at higher pH (≈12.0). In contrast, Subramonian et al. [26] reported better coagulation activity using *Cassia obtusifolia* seed gum (non-ionic polymer) under acidic conditions of pH 3 to 5, possibly due to slightly hydrolyzed organic contaminants promoting better adsorption on the gum.

For the sample of synthetic turbid water treated with the inorganic coagulant, turbidity and color removal between 37 and 46% were found at pH values between 1 and 7 (Figure 3a,b). This behavior was mainly attributed to the fact that ferric chloride is a cationic reagent (zeta potential = +9.56 ± 0.63 mV) and the surface of the synthetic turbid water was negatively charged, with electrostatic repulsion predominating, preventing effective floc formation [27]. However, synthetic turbid water at pH values between 8 and 13 had turbidity- and color-removal efficiencies between 52 and 94%, demonstrating the formation of large flocs. This behavior was attributed to the fact that at high pH, the positive charge of the iron coagulant may be neutralizing part of the negative charge on the surface of the particles in the synthetic turbid water, meaning that charge neutralization was the predominant mechanism [28].

This result means that the OFI fruit peel mucilage has the potential to be used as a coagulant reaching similar efficiencies to FeCl_3_ for turbidity and color removal in water treatment at a pH of 13 and a dose of 12 mg/L. For the biocoagulant the turbidity- and color-removal efficiency were 80+/− and 70+/−, respectively, and for the chemical coagulant the turbidity- and color-removal were 90+/− and 80+/−, respectively.

### 3.3. FTIR Analysis of Dried Flocs

**Figure 4:**
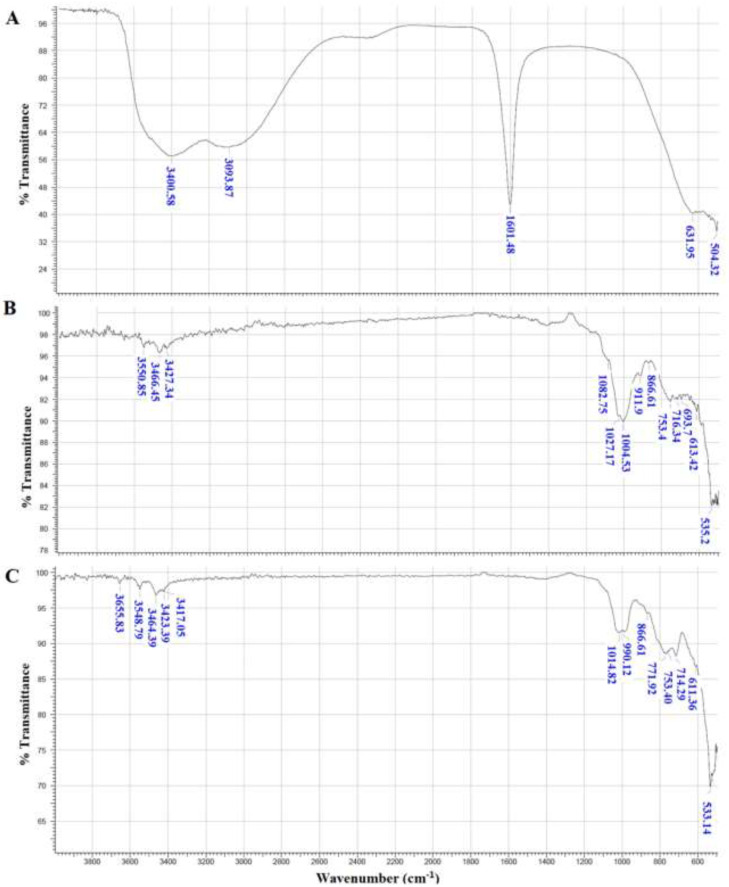
FTIR/ATR spectra in the range of 550 to 3900 cm^−1^ of the dry samples used in the coagulation/flocculation process. (**A**) Ferric chloride (FeCl_3_), (**B**) Flocs formed using the biocoagulant and (**C**) Flocs formed using the inorganic coagulant.

The FTIR spectra of the dried flocs formed from the coagulation/flocculation processes under optimal conditions, i.e., with a biocoagulant (OFI fruit peel mucilage) or chemical coagulant (ferric chloride) dosage of 12 mg/L and a pH of the turbid water of 13, are presented in Figure 4. For comparison, the FTIR spectra of the isolated coagulants (natural and synthetic) are also presented in Figure 4.

The main infrared absorbances observed in flocs formed with biocoagulant and chemical coagulant are attributable to the most representative functional and chemical groups present in mucilage, as we recently reported [4], and FeCl_3_ (Figure 4a) [26].

The FTIR spectrum of flocs formed from kaolin by biocoagulant, with defined peaks, (Figure 4b) showed a broad absorption band in the region of 3550–3427 cm^−1^ that was attributed to a stretching of the hydroxyl groups (O-H) indicating the presence of hydrogen bonding between hydroxyl groups adhered to the carboxylic terminals of the macromolecule galacturonic acid present in the polysaccharide chains [4] (reflects the anionic nature of mucilage) and the divalent cations present in the synthetic turbid water [2,27]. The bands observed within the range of 1082 to 866 cm^−1^ could be attributed to the stretching vibration of the C-O in C-OH of carbohydrates present in mucilage [29]. The main infrared absorbances observed in the flocs indicate that mucilage is adsorbing suspended solids present in synthetic turbid water due to the presence of hydroxyl and carboxyl functional groups [30].

The FTIR spectrum of flocs formed from kaolin by chemical coagulant (Figure 4c) showed a broad absorption band in the region of 3655–3417 cm^−1^ which was assigned to the -OH stretching vibrations indicating the presence of an electrostatic interaction between positive charges of the cationic chemical coagulant (that reflects the cationic nature of FeCl_3_) and the suspended negative charges in the synthetic turbid water [2,27]. Additionally, the strong and broad absorption centered at 1014 cm^−1^ with a shoulder at 886 cm^−1^ could be due to inorganic molecules’ absorptions [27]. Finally, both spectra evidence the interaction between the constituents of synthetic turbid water and coagulants (bio and chemical).

### 3.4. Zeta Potential of Flocs

The OFI fruit peel mucilage used as a biocoagulant in this study has a zeta potential of −23.63 ± 0.55 mV, which is consistent with its anionic nature [31]. Under optimal values (i.e., pH = 13 and coagulant dose = 12 mg/L), the flocs formed using this biocoagulant showed an increase in the absolute zeta potential (−37.10 ± 2.03 mV), indicating that coagulation occurs through an adsorption mechanism–particle bridge. A similar mechanism of coagulation was observed using OFI cladode mucilage in the pretreatment of water affected using the oil sand process [32]. In contrast, the chemical coagulant, with a zeta potential of +9.56 ± 0.63 mV (cationic behavior), led to the formation of flocs with a zeta potential of −10.19 ± 0.57 mV under optimal conditions, suggesting a coagulation mechanism of neutralization.

The zeta potential closest to zero (−10.19 ± 0.57 mV) for flocs formed from kaolin by chemical coagulation agrees with a neutralization mechanism promoted by electrostatic repulsion between kaolin and the chemical coagulant that causes colloidal destabilization, thus allowing the particles to agglomerate and favoring the flocculation process [33]. However, the low value of the zeta potential (−37.10 ± 2.03 mV) for the flocs formed by the biocoagulant can be attributed to the presence of NaCl in the synthetic turbid water which could affect the functional groups of the polysaccharides, thus influencing the bridging of kaolin particles with mucilage molecules [32]. This mechanism could affect the capacity and stability of the particles when flocs have formed [33].

### 3.5. Microscopic Morphology of Dried Flocs

**Figure 5:**
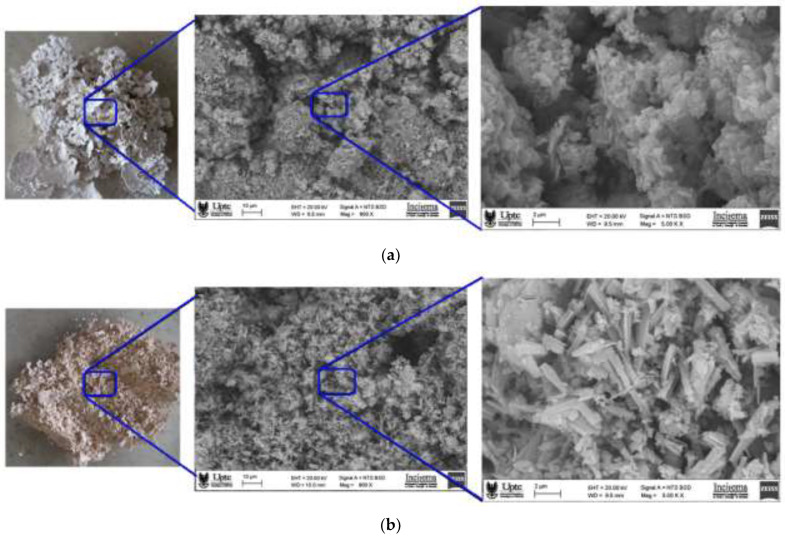
Macroscopic (left) and SEM (middle 800× and right 5000×) images of the dried flocs formed using biocoagulant (**a**) and chemical coagulant (**b**).

The SEM micrographs of the flocs obtained under a coagulant dose of 12 mg/L and at pH = 13 are shown in Figure 5.

As shown in Figure 5a, the floc structure obtained using biocoagulant captured at 800× magnification shows large flocs with coral-reef-like surfaces [34] and a smooth and compact appearance composed of fibrous networks. This morphology can be attributed to the formation of interparticle bridges. SEM images of the floc, which were taken at 5000× magnification, revealed thin, strong rough sheets, with cavities of irregular shape and size, a morphology that could be involved in the adsorption of colloidal particles from turbid water [35]. A similar morphology was observed for flocs formed with natural *Cassia obtusifolia* seed um in the removal of contaminants in agro-industrial wastewater [36].

The micrographic morphology of the flocs obtained using chemical coagulant, observed with a magnification of 800× (Figure 5b), also shows surfaces with a coral-reef-like appearance [34] with an irregular and dense structure and an agglomeration effect between the colloidal particles. Images of the floc taken at 5000× magnification revealed sheets with a rough, heterogeneous and loose surface with cavities in the form of scales. This morphology may be due to the complexation between particles of chemical coagulant and kaolin by a neutralization mechanism. A similar morphology was observed for flocs formed using FeCl_3_·6H_2_O in the removal of humic acid in wastewater [29].

## 4. Conclusions

This work demonstrated the effectiveness of the mucilage obtained from the peel of *Opuntia ficus-indica* fruit as a primary coagulant for the removal of turbidity and color in synthetic turbid water, results similar to those obtained using ferric chloride.

The optimal dose value for both coagulants in the coagulation/flocculation process was 12 mg/L at pH 13. Under these optimal conditions, the turbidity- and color-removal efficiency were for the biocoagulant 80+/− and 70+/− and for the ferric chloride 90+/− and 80+/−, respectively. This result is an indication of the potential use of the biocoagulant as an efficient alternative to chemical coagulants in water treatment, although pH adjustment will be required if the biocoagulant is used for the treatment of most natural waters. On the other hand, the analyses of the structure (FTIR and zeta potential) and morphology (SEM) of the flocs formed under optimal values revealed a coagulation mechanism using mucilage by adsorption and bridging attributed to the predominant presence of galacturonic acid in the polysaccharide chains; different from the case of the inorganic coagulant (FeCl_3_), which involved a charge-neutralization mechanism.

Finally, using a natural product as an alternative to inorganic coagulants for water clarification will facilitate potential biological management of the sludge generated during coagulation/flocculation. The foregoing adds to the potential for the use and recovery of an agricultural residue such as the peels of the *Opuntia ficus-indica* fruit.

## Data Availability

The data presented in this study are available on request from the corresponding author.
